# Relationship between oral health literacy and oral health status

**DOI:** 10.1186/s12903-018-0640-1

**Published:** 2018-10-24

**Authors:** Jagan Kumar Baskaradoss

**Affiliations:** Division of Dental Public Health, Department of Developmental and Preventive Sciences, Faculty of Dentistry, Kuwait University, P.O.Box: 24923, -13110, Safat, Kuwait

**Keywords:** Dental caries, Oral health, Literacy, Health literacy, Periodontitis, Periodontal disease

## Abstract

**Background:**

Health literacy has been found to be a strong predictor of an individuals’ health, health behavior and health outcomes. Lower literacy has been linked to problems with the use of preventive services, delayed diagnoses of medical conditions, poor adherence to medical instructions, poor self-management skills, increased mortality risks, poor health outcomes, and higher health care costs. The aim of this study is to determine the relationship between oral health literacy (OHL) and oral health status among patients attending a University-affiliated dental clinic.

**Methods:**

A convenience sample of participants was drawn from the dental patients presenting at School of Dental Medicine (SODM), Case Western Reserve University (CWRU). Information about the subjects’ demographic details, income, family size, insurance type and smoking history were collected using a data collection form. Data about the patients’ periodontal and caries risk assessment, caries experience and periodontal status was extracted from the patients’ electronic dental records. The Comprehensive Measure of Oral Health Knowledge (CMOHK) was used to record the oral health literacy. The median CMOHK score was 18, and this was used to categorize the sample into limited OHL (≤18) and adequate OHL (> 18) groups. A multivariate logistic regression model was built to examine the associations between the various independent variables and OHL levels.

**Results:**

Data of 150 respondents were analyzed. More than half of the participants were female (55.3%) and the majority were Caucasian (60%). The average age of participants was 53.3 years [standard deviation (SD) 16.8]. Higher percentage of African Americans and individuals with low education had limited OHL levels (*p* < 0.05). The mean decayed, missing, and filled teeth (DMFT) score for this population was 7.33 ± 2.68. Subjects with limited OHL had significantly higher mean values for missing teeth (*p* < 0.05) and lower mean values for filled teeth (*p* < 0.05) as compared with subjects with adequate OHL Significantly, higher percentage of subjects with limited OHL had severe periodontitis as compared with those with adequate OHL (*p* = 0.04). Multivariate analysis found that the periodontal status was significantly associated with the OHL scores (*p* = 0.015).

**Conclusion:**

Subjects with limited OHL levels had poorer periodontal health. Improving the OHL of patients may help in the efforts to improve the adherence to medical instructions, self-management skills and the overall treatment outcomes. Future research could focus on assessing the impact of OHL interventions on the oral health, which could be valuable for clinical practitioners.

## Background

Health literacy is “the degree to which individuals have the capacity to obtain, process, and understand basic health information and services needed to make appropriate health decisions [1].” This includes the ability to read and understand written text, to effectively communicate health-related information, to navigate the healthcare system and to attain and maintain good health.

An individual’s health literacy capacity is mediated by education, and its adequacy is affected by culture, language, and the characteristics of health-related settings. Health literacy has been found to be a strong predictor of an individuals’ health, health behavior and health outcomes [2, 3]. Limited health literacy is associated with poor self-ratings of health, poor adherence to medical instructions, poor self-management skills, increased mortality risks, poor health outcomes, and higher healthcare costs [4–6]. In the US, the National Assessment of Adult Literacy Survey reported that nearly half (43%) of adults in the United States (U.S.) are at risk for low literacy [7]. Health literacy is now recognized as an underlying cause of health disparities and has become a national health priority [8, 9]. World Health Organization’s (WHO’s) 7th Global Conference on Health Promotion also list’s health literacy as one of the five key tracks for promoting health [10] Oral health literacy (OHL) has gained prominence in the dental literature in the last decade. Similar to health literacy, OHL has also proved to be critical in reducing oral health disparities and in promoting oral health [11]. Individuals with limited OHL were reported to be at higher risk for oral diseases and the problems related to those diseases [12]. Lower literacy has been linked to problems with the use of preventive services, delayed diagnoses of medical conditions, poor adherence to medical instructions, poor self-management skills, increased mortality risks, poor health outcomes, and higher health care costs [5]. Baskaradoss [13] found that people with poor oral health literacy are more likely to have missed dental appointments. Non-adherence to dental recommendations has been reported to cause higher caries experiences [14] and poor periodontal status [15]. Several publications from the Carolina Oral Health Literacy (COHL) study [16], have highlighted the important role played by OHL in influencing health behaviors and health outcomes [16–19]. In a large cross-sectional survey conducted at two university-based dental clinics in the United States, Multi-site Oral Health Literacy Research Study (MOHLRS), reported that about one-fifth (18%) of the participants had “low” conceptual knowledge [20]. Ju et al. [21] conducted an interventional study among Indigenous Australian adults, where context-specific oral health literacy intervention was successful in improving the oral health literacy-related outcomes.

The relationship between OHL and health outcomes has been explained by Macek et al. through their conceptual model [22]. A person’s health is a consequence of the health related decisions made by them, which in turn is influenced by health literacy, modulated by the various sociodemographic factors. According to the conceptual model, health determinants such as income, education and personal characteristics influence health behaviors and oral health outcomes.

The purpose of this study was to explore the relationship between OHL and oral health status among patients attending a University-affiliated dental clinic.

## Methods

A convenience sample of participants was drawn from patients of record presenting at School of Dental Medicine (SODM), Case Western Reserve University (CWRU), Ohio, USA, from February through April of 2015. Written informed consent and Health Insurance Portability and Accountability Act (HIPAA) consent forms were obtained for study participation. The research was conducted in accordance with the World Medical Association Declaration of Helsinki and approved by the CWRU Institutional Review Board (IRB) (Protocol number: IRB-2014-1003).

This study included African-American or Caucasian patients who were at least 18 years old and had the ability to provide informed consent to participate in the study. Patients who required emergency care were excluded from the study. A single trained investigator collected the data. The subjects were informed that their participation was voluntary and were assigned a separate scheduled cubicle for completing the questionnaires. Information about the subjects’ demographic details, income, family size, insurance type and smoking history were collected using a data collection form. Data about the patients’ periodontal and caries risk assessment were extracted from the patients’ electronic dental records. The caries and periodontal charting and risk assessments are routinely performed for all patients and are based on the risk assessment–based individualized treatment model. The components of this risk assessment were published previously [23]. Indicators for dental caries were calculated based on the number of decayed, missing, and filled teeth (DMFT) as proposed by Klein et al [24]. Proximal caries was confirmed using radiographs, which are routinely taken for all the patients.

This study focused on patients with periodontitis as defined by the Centers for Disease Control and Prevention (CDC) [25], which defines disease as following: Severe Periodontitis: ≥2 interproximal sites with CAL ≥6 mm (on more than one tooth) and ≥ 1 interproximal site with PD ≥5 mm; Moderate Periodontitis: ≥2 interproximal sites with CAL ≥4 mm(on more than one tooth) or ≥ 2 interproximal sites with PD ≥5 mm (on more than one tooth); Healthy or Mild Periodontitis: neither “moderate” nor “severe” periodontitis. The Comprehensive Measure of Oral Health Knowledge (CMOHK) was used to record the oral health literacy of the respondents [22].

Previous studies on OHL in a similar University hospital setting have established the minimum sample size of 102 to detect enough power at α = 0.05 [15, 26]. Therefore, a sample size of 150 in this study was considered adequate. The responses were entered into the Statistical Package for the Social Sciences (SPSS 22.0; SPSS Inc., Chicago, IL, USA) for Windows. Exploratory analyses were performed to examine the distributions of the data and to identify outliers and missing data. Bivariate analyses were used to explore the associations between each of the covariates and OHL via Pearson’s χ^2^ statistics for categorical variables and Mann-Whitney U Test for continuous variables. The missing values for some of the variable were imputed using mean substitution method.

The CMOHK scores were negatively skewed and hence, nonparametric analyses were performed. A multivariate logistic regression model was built using the ‘Enter’ method to examine the associations between the demographic characteristics, dental risk factors, oral health indicators and OHL levels. The median CMOHK score was 18, and this was used to categorize the sample into limited OHL (≤18) and adequate OHL (> 18) groups.

## Results

Of the 174 patients invited for this study, 7 refused to participate, and 17 provided incomplete responses that were discarded. Therefore, the data from 150 respondents were included in the analysis. More than half of the participants were female (55.3%) and the majority were Caucasian (60%). The average age of participants was 53.3 years [standard deviation (SD) 16.8]. Table 1. shows the socio-demographic characteristics of the participants. More than a quarter (26%) of the participants had finished high school or received a General Equivalency Diploma (GED) or less, 34% had attended or completed community college, 29.3% had attended or completed college, and 10.7% had a professional degree. The majority of the participants were above the Federal Poverty Level (FPL) (74%) and paid for their dental treatments with cash (41.3%). Bivariate comparisons suggest that race and education levels were significantly associated (*P* < 0.05) with OHL levels. Higher percentage of Caucasians and educated individuals had adequate OHL as compared to others. Majority (93.3%) of the participants were nonsmokers (Table 2).

**Table 1:** Distribution of Socio-demographic variables by OHL levels

Variables	All Subjects	Low OHL	High OHL	*p*-value*
	N (%)	(<=18) N (%)	(> 18) N (%)	
Mean age ± SD	53.3 ± 16.8	54.8 ± 16.7	50.8 ± 16.8	0.79†
Gender				
Male	67 (44.7)	44 (47.8)	23 (39.7)	0.327
Female	83 (55.3)	48 (52.2)	35 (60.3)	
Race				
African-Americans	59 (39.3)	42 (45.7)	17 (29.3)	0.046
Caucasians	91 (60.7)	50 (54.3)	41 (70.7)	
Education				
High school graduate/GED or less	39 (26.0)	34 (44.6)	5 (8.6)	0.004
Some college or technical degree	51 (34.0)	28 (30.4)	23 (39.7)	
College degree	44 (29.3)	23 (25.0)	21 (36.2)	
Professional degree	16 (10.7)	7 (7.6)	9 (15.5)	
Payment Type				
Public/Private insurance	88 (58.7)	57 (62.0)	31 (53.4)	0.303
Out-of-pocket	62 (41.3)	35 (38.0)	27 (46.6)	
Socio-economic Status				
Below FPL	39 (26.0)	29 (31.5)	10 (17.2)	0.052
Above FPL	111 (74.0)	63 (68.5)	48 (82.8)	
Marital Status‖				
Single	47 (35.9)	31 (38.3)	16 (32.0)	0.46
Married	78 (59.5)	45 (55.6)	33 (66.0)	
Divorced/widow	6 (4.6)	2 (6.1)	4 (2.0)	

*FPL* Federal poverty level, *GED* General equivalency diploma, *OHL* Oral health literacy; *Chi-squared test; †Independent samples T-Test; ‖Presence of missing values

**Table 2:** Distribution of oral health characteristics by OHL levels

Variables	All Subjects	Low OHL	High OHL	*p*-value*
	N (%)	(<=18) N (%)	(> 18) N (%)	
Cigarette Smoking				
Current Smoker	10 (6.7)	4 (4.3)	6 (10.3)	0.137
Former/Never Smoker	140 (93.3)	88 (95.7)	52 (89.7)	
Caries risk level‖				
Low	20 (13.3)	12 (13.0)	8 (13.8)	0.67
Moderate	56 (37.3)	34 (37.0)	22 (37.9)	
High	67 (44.7)	39 (42.4)	28 (48.3)	
Extremely high	3 (2.0)	3 (3.3)	0 (0.0)	
Periodontal risk level‖				
Low	50 (33.3)	27 (29.3)	23 (39.7)	0.006
Moderate	64 (42.7)	34 (37.0)	30 (51.7)	
High	31 (20.7)	27 (29.3)	4 (6.9)	
Periodontitis				
Healthy/Mild	68 (45.3)	36 (39.1)	32 (55.2)	0.039
Moderate	42 (28.0)	25 (27.2)	17 (29.3)	
Severe	40 (26.7)	31 (33.7)	9 (15.5)	
Caries Experience				
DT	0.84 ± 0.95	0.95 ± 0.93	0.67 ± 0.96	0.561†
MT	2.93 ± 2.07	2.95 ± 1.87	2.90 ± 2.37	0.004†
FT	3.6 ± 2.1	3.29 ± 1.91	3.98 ± 2.33	0.019†
DMFT	7.33 ± 2.68	7.18 ± 2.54	7.55 ± 2.89	0.281†

*OHL* Oral health literacy, DMFT is number of decayed, missing, and filled permanent teeth, DT is number of decayed permanent teeth, MT is number of permanent teeth missing due to disease, and FT is number of filled permanent teeth

* Chi-squared test; † Mann-Whitney U Test; ‖Presence of missing values

The mean DMFT for this population was 7.33 ± 2.68. There was no significant difference between the two groups in terms of the decay score or the overall DMFT scores. Majority (60.9%) of the subjects had limited OHL. Conversely, subjects with limited OHL had significantly higher mean values for missing teeth (*p* < 0.05) and lower mean values for filled teeth (p < 0.05) as compared with subjects with adequate OHL. There was no difference in the caries risk level between the 2 groups. However, there was statistically significant difference between subjects with limited and adequate OHL in relation to the periodontal risk assessment levels. More than a third of the subjects with limited OHL had high periodontal risk levels as compared with only about 7% of subjects with adequate OHL. Periodontitis was distributed as 45.3%, 28.0%, and 26.7% with mild/healthy, moderate and severe periodontitis, respectively. Similarly, higher percentage of subjects with limited OHL had severe periodontitis as compared with those with adequate OHL, which was statistically significant (*p* = 0.04). Various dental covariates were included using the Enter method for the Multivariate analysis (Table 3). The final model included the following variables: Caries risk assessment, Periodontal risk assessment, DMFT scores and Periodontal status. Periodontal status was found to be significantly associated with the OHL scores (*p* = 0.015).

**Table 3:** Multivariate logistic regression model of dental characteristics and OHL scores

	*p*-values	Exp(B)	95% C.I. for EXP(B)	
			Lower	Upper
DMFT	0.254	1.079	0.947	1.231
Periodontitis	0.015	0.579	0.372	0.901
PRA_New	0.378	0.703	0.322	1.538
CRA_New	0.447	1.543	0.505	4.714
Constant	0.829	0.785		

*DMFT* Decayed missing filled teeth, *CRA* Caries risk assessment and *PRA* Periodontal risk assessment

## Discussion

In this study, there was significant associations between several oral health characteristics and the OHL levels. Those with low OHL had the highest risk for oral diseases and the problems related to those diseases. Health literacy is a known mediator between socio-economic factors, health behavior and oral health outcomes in various populations, explaining gradients in oral health status and outcomes [3].

This study included only African-American or Caucasian patients as they constitute a majority (approx.90–95%) of the patient population seen at the dental clinic. Disparities in OHL levels by race/ethnicity and by socioeconomic status (SES) have been widely documented. This study also found significant difference between race and educational levels with levels of OHL. Higher percentage of African Americans and individuals with low education have limited OHL levels. Similar findings have been reported previously in other studies [26, 27]. These disparities in oral health have been attributed to a complex web of social, psychological, and structural factors, such as nutrition, oral hygiene, healthcare utilization, and access to care [28]. OHL has proved to be critical in reducing oral health disparities and in promoting oral health [11].

The mean DMFT for this population was about half the National average as reported in the National Health and Nutrition Examination Survey (NHANES), 1999–2004 [29]. The could be due to the difference in the population studied. This study was conducted in a University hospital setting as compared with the community based NHANES survey. In a study by Blizniuk et al. [30], participants with adequate oral health literacy had fewer missing and more filled teeth than those with inadequate literacy. This is similar to the findings of this study. A possible explanation is that an individual with adequate OHL not only recognizes oral diseases at an earlier stage than someone with limited OHL, but also is more prompt in seeking the required treatment. Individuals with limited OHL are often more prone to delayed diagnoses of one’s dental conditions which is explained by the higher percentage of missing teeth in this group.

The distribution of periodontitis in this study was markedly different from the national average as reported in the NHANES data (mild - 8.7%, moderate - 30.0%, and severe - 8.5%) [31]. This again could be due to the difference in the population studied and also due to the difference in the criteria used in defining periodontitis. Periodontal disease is a chronic disease, therefore, the patients understanding and compliance are essential for successful long-term maintenance and periodontal stability [32]. In this study, subjects with limited OHL levels had higher prevalence of severe periodontitis. This is in contrast to the findings of the study by Wehmeyer et al. [15]. A possible explanation for this discrepancy could be the difference in the instrument used to record OHL. Wehmeyer et al. [15] used the Rapid Estimate of Adult Literacy in Dentistry (REALD) - 30 for assessing the OHL. However, REALD − 30 does not have the specificity to assess the subjects’ knowledge levels pertaining to periodontal health. Holtzman et al., [33], reported significant association between OHL (as measured with Rapid Estimate of Adult Literacy in Medicine and DentistryREALMD-20 and CMOHK) and clinical measures of periodontal health. CMOHK consists of questions that measures general oral health knowledge, as well as specific questions assessing the knowledge of oral conditions like caries, periodontal diseases and cancer. Though CMOHK was initially considered to measure only the oral health conceptual knowledge, recent studies have been able to support the contention that conceptual knowledge is, indeed, a construct of health literacy. In a recent study by Macek et al. [20], it was reported that CMOHK scores were significantly associated with the scores of other health literacy instruments like the Rapid Estimate of Adult Literacy in Medicine (REALM) and Short-test of functional health literacy in adults (TOFHLA), thus confirming CMOHK’s validity. In the original study [22], the CMOHK scores were divided into the following three categories: poor (0–11), fair (12–14), and good (15–23). However, the oral health literacy scores in this study were significantly higher (mean = 16.7); hence, the scores were dichotomized based on the median score of 18. The difference in the scores between the two studies can be attributed to differences in the sample populations as described in an earlier study [13].

The present results should be considered in light of the study’s limitations. Firstly, the data were collected from a nonprobability convenience sample of patients from a university-based dental clinic. This study reflects the health-seeking behaviors of patients attending a university-based dental clinic only and not necessarily that of the community. The cross-sectional design of this study prevents it from elaborating on the cause and effect. Further longitudinal studies or clinical trials may be required to extend the findings reported here. The other limitation of this study is that all the clinical measurements were obtained from the electronic records and not taken directly by the investigator. However, this may not impact the validity of the data since the measurements are routinely checked by a trained specialist before they are entered in the electronic database.

## Conclusion

In conclusion, subjects with limited OHL levels had poorer periodontal health. Improving the OHL of patients may help in the efforts to improve the adherence to medical instructions, self-management skills and the overall treatment outcomes.

## Abbreviations

CDC: Centers for Disease Control and Prevention
CMOHK: Comprehensive Measure of Oral Health Knowledge
CRA: Caries Risk Assessment
CWRU: Case Western Reserve University
DMFT: Decayed, missing, and filled teeth
FPL: Federal Poverty Level
GED: General Equivalency Diploma
HIPAA: Health Insurance Portability and Accountability Act
IRB: Institutional Review Board
NHANES: National Health and Nutrition Examination Survey
OHL: Oral Health Literacy
PRA: Periodontal Risk Assessment
SES: Socioeconomic status
SODM: School of Dental Medicine
